# Identification of microRNA Transcriptome Involved in Bovine Intramuscular Fat Deposition

**DOI:** 10.3389/fvets.2022.883295

**Published:** 2022-04-15

**Authors:** Susan K. Duckett, Maslyn A. Greene

**Affiliations:** Department of Animal and Veterinary Sciences, Clemson University, Clemson, SC, United States

**Keywords:** beef, marbling, microRNA, mRNA, intramuscular fat content

## Abstract

**Background:**

Intramuscular fat deposition in beef is a major determinant of carcass quality and value in the USA. The objective of this study was to examine changes in microRNA (miRNA) transcriptome that are involved with intramuscular fat deposition with time-on-concentrates (TOC). Yearling steers were individually fed a high concentrate diet and changes in intramuscular fat deposition were monitored by real-time ultrasound at 28 to 33 d intervals. Longissimus muscle biopsies collected on d 0, 92 and 124 TOC to examine changes in miRNA transcriptome that are involved in intramuscular fat deposition.

**Results:**

Steer body weight increased (*P* < 0.0001) at each weigh day during TOC. Fat thickness increased (*P* < 0.005) from d 28 to 124. Ribeye area was larger (*P* < 0.001) on d 124 than d 61, which was larger than d 0 and 28. Ultrasound intramuscular fat content was greater (*P* < 0.001) on d 92 and 124 compared to d 0, 28 or 61. Sequencing of the muscle biopsy samples identified one miRNA, bta-miR-122, that was up-regulated (*P* < 0.005) at d 92 and 124 compared to d 0. At d 92 TOC, mRNA expression levels of fatty acid binding protein 4 (FABP4) and elongase 6 (ELOVL6) were up-regulated (*P* < 0.01) compared to d 0; whereas at d 124, lipogenic genes involved in *de novo* fatty acid synthesis, fatty acid transport, elongation and desaturation were highly up-regulated compared to d0.

**Conclusions:**

Small RNA sequencing identified bta-miR-122 as a potential miRNA of interest that may be involved in intramuscular fat deposition with increasing TOC. Increased intramuscular fat content, as measured by real-time ultrasound, combined with differential gene expression suggests that preadipocyte differentiation may be stimulated first, which is followed by a global up-regulation of lipogenic genes involved in *de novo* fatty acid synthesis that provide fatty acids for subsequent hypertrophy.

## Introduction

Marbling or intramuscular fat deposition in beef is a major determinant of carcass quality and value in the USA. Consumer demand for US Prime and branded Choice beef products is at an all-time high but only about 4% of carcasses reach the Prime quality grade (1). Beef producers are working to select genetics with higher genetic ability to marble and evaluating management systems that help produce a greater percentage of carcasses into the premium quality grades. Current premiums for carcasses grading Prime with a yield grade of 1 to 3 are $0.52/kg carcass weight or an additional $208 premium for a 400 kg carcass (2). Serial slaughter studies indicate that marbling deposition increases after 80 d on a high concentrate diet (3–6). Feeding high concentrates to steers upregulates key lipogenic genes and marbling deposition in early weaned calves (7, 8), normal weaned calves (9, 10) and yearling calves (11). Duckett et al. (3) found that intramuscular fat deposition doubled in the longissimus muscle between 86 and 112 d on concentrates with no change after that when fed for a total of 196 d on concentrates.

The transient increases in intramuscular fat deposition suggest that epigenetic adaptation may be playing a role in this process. microRNA (miRNA) are small, non-coding RNAs that play a role in post-transcriptional gene regulation. Research shows that miRNA expression differs between depot (12, 13) and finishing systems in beef cattle (12) and sheep (14). Others have identified specific miRNAs that can inhibit differentiation of ovine stromal vascular cells = (15). In a review article, Romao and co-workers (16) concluded that miRNA are involved in the adipogenic process in farm animals and that more research was needed to identify specific miRNA that regulate fat deposition. We hypothesize that key miRNAs are involved with increased intramuscular fat deposition with TOC. The objective of this study was to identify key miRNAs and changes in mRNA expression that coincide with enhanced intramuscular fat deposition during TOC.

## Materials and Methods

Experimental procedures were reviewed and approved by Clemson University Animal Care and Use Committee, AUP2020-001.

### Animals

Angus-cross, yearling steers (*n* = 7; 428 ± 22 kg BW; 12.3 mo of age) were selected from Clemson University Piedmont Research and Education Center. All steers were sired by the same Angus bull (Connealy Mentor 7374, +0.73 marbling EPD, 0.67 accuracy). Steers were weighed on consecutive days at the beginning and end of the experiment. Steers were individually fed using Calan gates and adjusted to high concentrate ration using step-up rations (Table 1). Steers were weighed and ultrasounded at about 28–33 d intervals during the study. A biopsy of longissimus muscle (LM) was obtained on d 0, 92 and 124 on concentrates. Steers were finished for 124 d (16.6 mo of age) and transported to a commercial packing plant for slaughter. Due to the Covid-19 pandemic, we were unable to enter the packing plant and obtain actual carcass data for this study.

**Table 1:** Composition of the high concentrate diet fed to steers in this study.

**Ingredient**	**Step 1^a^**	**Step 2^a^**	**Final^a^**
CPC Grower, %	50	33	0
Corn, rolled, %	40	54	80
Soybean meal, %	4.5	6.0	9
Limestone, %	0.25	0.3	0.5
Trace mineral pre-mix with Rumensin^b^, %	0.25	0.3	0.5
Oat hay, ground, %	5	6.7	10
**Nutrient Composition**			
Crude protein, %	13.2	13.3	13.4
NEm, mcal/kg			2.07
NEg, mcal/kg			1.40

a *Step1 and Step 2 rations were fed for 7 d each and then they consumed the final ration from d 15 to 124*.

b *Trace mineral premix composition: calcium, 0.67%; phosphorus, 0.33%; magnesium 0.15%; potassium, 0.56%; sulphur, 0.17%; copper, 22.78 mg/kg; manganese 72.49 mg/kg; selenium, 0.40 mg/kg; zinc, 98.12 mg/kg; monensin, 38.79 mg/kg*.

### Real-Time Ultrasound

Real-time ultrasound measures of fat thickness, ribeye area and intramuscular fat percentage were collected between the 12th- and 13th-ribs using an Aloka 500-V ultrasound (Corometrics Medical Systems, Wellingford, CT) equipped with a 17-cm, 3.5-MHz linear probe. The images were interpreted using Biosoft Toolbox (Biotronics, Inc., Ames, IA).

### Muscle Biopsy

Longissimus muscle needle biopsies were obtained on each steer at 0, 92 and 124 d on concentrate diet. Steers were briefly restrained in a mechanical chute and the LM from the 10^th^ rib to 13th rib region was shaved. A Bergstrom biopsy needle (Millennium Surgical, Bala Cynwyd, PA) was used to obtain about 200 mg from the center of the longissimus muscle. Muscle biopsy samples were trimmed of any external subcutaneous fat or epimysial connective tissue and then immediately frozen in liquid nitrogen. Biopsy samples were transported to Clemson University in liquid N_2_ and stored at −80°C until extraction. Skeletal muscle biopsy locations were taken at 11th rib on the left side (d 0), 11th rib on right side (d 92) and 12th rib on left side (d 124) to avoid sampling in a remodeled area.

### RNA Extraction

Total RNA was isolated from the muscle biopsy samples using the TriZol procedure (Invitrogen; Thermo-Fisher, Waltham, MA). RNA extracts were DNased (DNA-Free DNA removal kit, ThermoFisher). Total RNA was quantified using a NanoDrop One spectrophotometer (ThermoFisher) and quality assessed using Agilent Bioanalyzer (Agilent, Santa Clara, CA). The RNA integrity number (RIN) for all samples was 7 or greater.

### miRNA Sequencing

RNA samples (*n* = 12; 4 steers x 3 TOC/steer) were shipped on dry ice to PrimBio (Exton, PA) for small RNA sequencing. The four steers selected for the miRNA sequencing were closest to the treatment mean for IMF content at d 92 and d 124. Small RNA was enriched with the Total RNA-seq Kit v2 (ThermoFisher) according to manufacturer. Small RNA samples were run on an Agilent 2100 Bioanalyzer to assess yield and size distribution of the Small RNAs. cDNA libraries were constructed using Ion Total RNA-Seq Kit v2 (4479789; ThermoFisher). Small RNA (30 ng) was hybridized with Ion adapters in a thermocycler for 10 min at 65°C and 5 min at 16°C. Hybridized Small RNA was then incubated overnight at 16°C with ligation enzyme mix to ligate the Ion adapters. Hybridized samples were then mixed with a reverse transcriptase master mix and incubated at 70°C for 10 min, snap cooled, and incubated 42°C for 30 min to generate cDNA libraries. cDNA libraries were purified using nucleic acid binding beads and buffers according to the manufacturer protocol (Magnetic Bead Cleanup Module, 4479789, ThermoFisher). The purified cDNA libraries were amplified by PCR using Platinum PCR Super-Mix High Fidelity and indexed bar codes added with Ion Xpress RNA Barcode reverse and forward primers according to manufacturer. The quality of each final library was assessed using the Agilent^®^ dsDNA High Sensitivity Kit.

Approximately 50 pM of pooled barcoded libraries were used for templating using Thermo Fisher Ion 540 CHEF Kit (A30011) according to the manufacturer's protocol. Samples were assessed for polyclonal percentage using a Qubit 4 (Invitrogen, ThermoFisher). Samples were then loaded onto a 540 chip, placed into an Ion S5 sequencer, and run using an Ion Torrent Small RNAseq run plan. After completion of the proton run, the raw sequence files (fastq) were aligned to the bovine genome (bosTau4) reference sequences by the StrandNGS software using the default parameters. Aligned SAM files were used for further analysis. Quality control was assessed by the Strand NGS program, which determined the pre- and post-alignment quality of the reads for each sample. The aligned reads were normalized and quantified using the Quantile algorithm by the StrandNGS program. The total raw reads generated by sequencing was 87,994,733 with a minimum of 5,194,893 reads per individual sample and all samples had a Q20 of > 90%. Average read length was 22 nucleotides. Statistical analysis was performed using the ANOVA test to determine significant differentially expressed small RNAs by time-on-concentrate. After significant small RNAs were identified, significant fold change was determined and small RNAs that had a significant fold change of 1.3x or higher were identified.

### miRNA qPCR Validation

TaqMan miRNA reverse transcription kit (catalog no = 4366597, ThermoFisher) was used to convert miRNA to cDNA. TaqMan Small RNA Assay kit was used for has-miR-122-5p (catalog no = 4427975, assay no = 002245; ThermoFisher), which has the same sequence as bta-miR-122 (Table 2). Relative gene expression of miR-122 was analyzed using ANOVA by TOC (d0, 92, and 124) using GraphPad Prism 9.3.1 (18).

**Table 2:** miR-122 sequence alignment for *Bos taurus* (bta), *Ovis aries* (oar), and *Homo sapiens* (hsa).

miR-122 Mature Sequence (miRbase)	
bta-miR-122 (MIMAT0003849)	UGGAGUGUGACAAUGGUGUUUG
oar-miR-122 (17)	UGGAGUGUGACAAUGGUGUUUG
hsa-miR-122-5p (MIMAT0000421)	UGGAGUGUGACAAUGGUGUUUG
TaqMan assay hsa-miR-122-5p^1^	UGGAGUGUGACAAUGGUGUUUG

*TaqMan Small RNA Assay kit, catalog no = 4427975, assay no = 002245 (ThermoFisher)*.

### mRNA qPCR

RNA (1 ug) was reverse transcribed using qScript (QuantaBio, VWR) for qPCR (QuantStudio3 Real-Time PCR system, Applied Biosystems) using SYBR green (PerfeCTa SYBR Green SuperMix; QuantaBio) according to the manufacturer. Primers for genes involved in adipogenesis and lipogenesis were developed using PrimerQuest Tool (IDT, Coralville, IA) and sequences are listed in Supplementary Table 1. Several housekeeping genes (glyceraldehyde 3-phosphate dehydrogenase [GAPDH], β-actin [ACTB], ubiquitously expressed prefoldin like chaperone [UXT], and eukaryotic translation initiation factor 3 subunit K [EIF3K]) were evaluated using RefFinder, https://www.heartcure.com.au/reffinder/, (19) to identify that most stable housekeeping gene(s). The most stable housekeeping genes were EIF3K (comprehensive stability index, M = 1.86) and UXT (M = 2.28). The geometric mean of EIF3K and UXT was calculated (M = 1.19) and used for data normalization (20). Relative gene expression was calculated using the mean ΔC_T_ of the d 0 values and subjected to ANOVA to determine statistical differences by TOC using GraphPad Prism 9.3.1 (18).

### Statistics

Data were analyzed in a completely randomized design using the Mixed procedure of SAS (SAS Inst. Inc., Cary, NC) with time-on-concentrate in the model. Steer was the experimental unit. Least square means were generated and separated using a protected least significant difference test. Significance was determined at *P* < 0.05.

## Results

**Figure 1:**
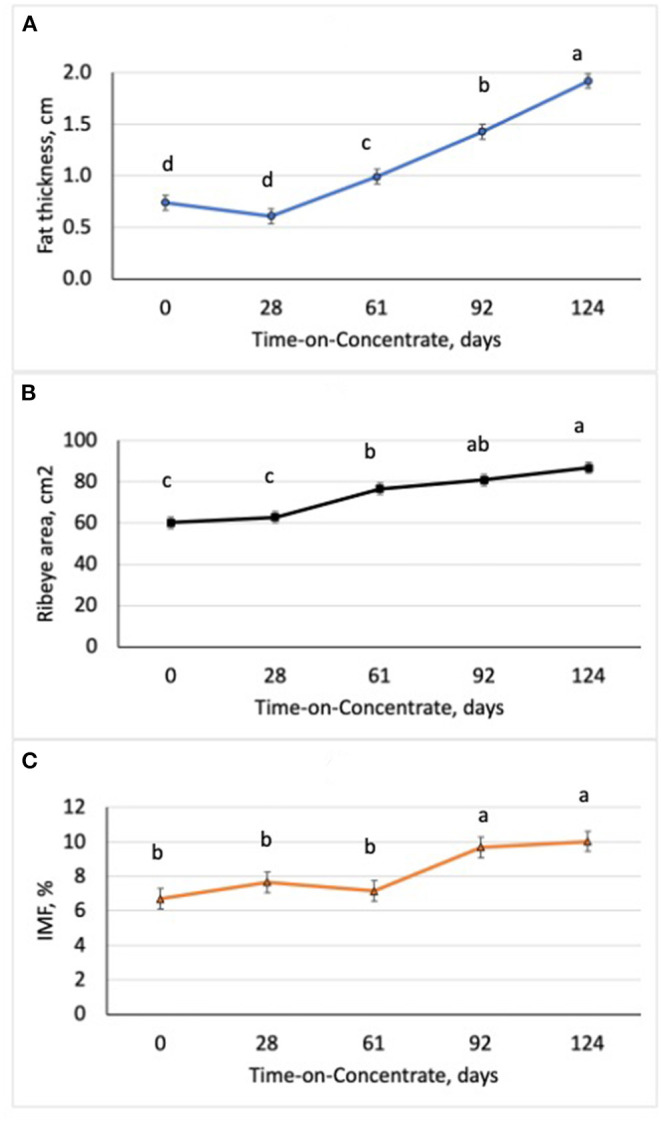
Changes in ultrasound fat thickness **(A)**, ribeye area **(B)**, and intramuscular fat content (IMF; **C**) in steers (*n* = 7/time) with increasing time-on-concentrates. ^abc^Means with uncommon superscripts differ (*P* < 0.01) for each graph.

Performance of the steers across TOC for this study is shown in Table 3. Steer body weight (BW) increased (*P* < 0.0001) at each time of measurement (28–33 d periods) during TOC. Steers finished with an average 659 kg body weight (BW). Average daily gains were greatest (*P* < 0.0001) during period 2 (d 29–61) and lowest (*P* < 0.0001) during period 1 (d 0–28). Dry matter intake was greater (*P* < 0.0001) during period 3 (d 62–92) and 4 (d 93–124) compared to period 1 and 2. Dry matter intake was also greater (*P* < 0.0001) for period 2 than period 1. Feed efficiency was greater (*P* < 0.0001) during periods 1 and 2 than periods 3 and 4 due to higher gains and lower feed intake during the first half of the TOC feeding. Changes in subcutaneous fat deposition, ribeye area, and intramuscular fat deposition were measured during time-on-concentrates using real-time ultrasound to estimate carcass parameters. Fat thickness was similar between d0 and d28 TOC (Figure 1A); however, fat thickness increased (*P* < 0.001) at each TOC from d28 to 124 d. Ribeye area was larger (*P* < 0.001) on d 124 than d 61, which were larger than d 0 and 28 (Figure 1B). Ultrasound intramuscular fat content did not differ from d 0 to d 61 but was greater (*P* < 0.001) on d 92 and d 124 than d 0, 28 or 61 TOC (Figure 1C).

**Table 3:** Live weight, average daily gain (ADG), dry matter intake (DMI) and feed efficiency (Gain:Feed) for the steers fed concentrates over time.

	**Live weight, kg**		
d 0	428.2^e^		
d 28	464.2^d^		
d 61	546.8^c^		
d 92	611.7^b^		
d 124	659.4^a^		
SEM	8.79		
*P*-Level	0.0001		
	**ADG, kg/d**	**DMI, kg/d**	**Gain:Feed**
Period 1 (d 0–28)	1.28^c^	5.50^c^	0.228^a^
Period 2 (d 29–61)	2.50^a^	11.44^b^	0.220^a^
Period 3 (d 62–92)	2.09^ab^	14.09^a^	0.149^b^
Period 4 (d 93–124)	1.66^bc^	13.33^a^	0.124^b^
SEM	0.181	0.493	0.0158
*P*-Level	0.0001	0.0001	0.0001

**abcde:** *Means in the same column differ (P < 0.05) for that variable*.

**Figure 2:**
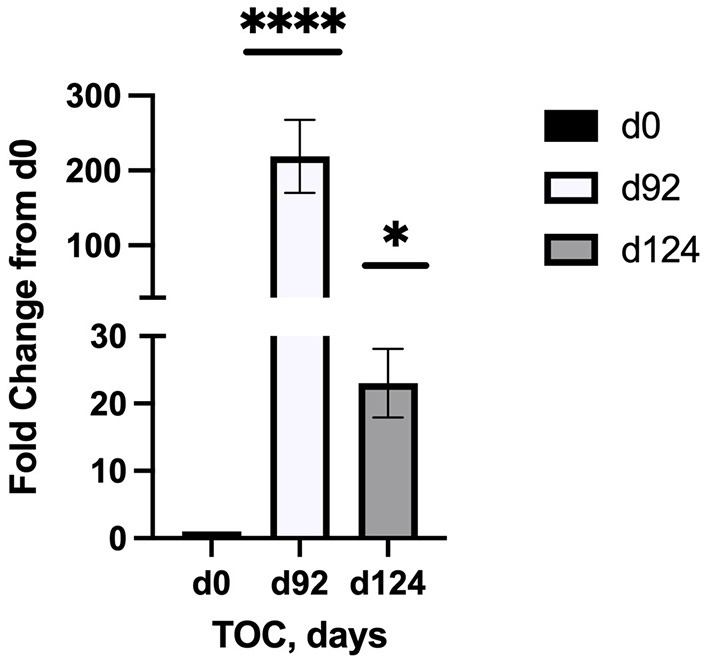
Fold change in miR-122 expression of skeletal muscle biopsies (*n* = 4/time) at d 92 and d 124 on concentrates compared to d0. Differences are noted (*P < 0.05; **P < 0.01; ***P < 0.001; ****P < 0.0001) in comparison to d 0.

**Figure 3:**
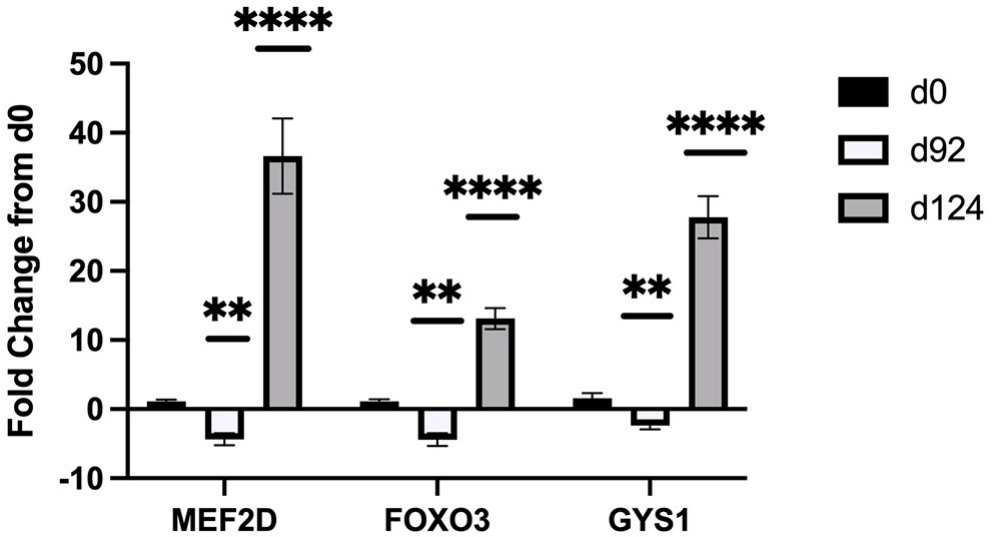
Fold change in mRNA expression of miR-122 targets (MEF2D, FOXO3, GYS1) at d 92, and d 124 compared to d 0 (n = 4/time). Differences are noted (*P < 0.05; **P < 0.01; ***P < 0.001; ****P < 0.0001) in comparison to d 0.

Sequencing of the muscle biopsy samples identified one miRNA, bta-miR-122, that was up-regulated (*P* < 0.005) by 6.72-fold on d 92 compared to d 0 (Table 4). Several miRNA were down-regulated (*P* < 0.05) at d 92 by −1 to −2 fold, which included miR-323, −449a, −1197,−485, and−2411. At d 124, 8 miRNAs were up-regulated (*P* < 0.05) and 7 were down-regulated (*P* < 0.05) compared to d 0 (Table 5). miR-122 was up-regulated (*P* < 0.05) at d 124 by 3.8-fold compared to d 0. Other miRNAs that were up-regulated included miR-383,−2346,−144,−505,−142,−1248-1 and−1248-2. The down-regulated miRNAs included miR-196b,−208b,−196a-1,−326-5p,−196a-2,−449a, and−2411 from d 124 compared to d 0. The mature sequence of bta-miR-122 is identical to ovine (oar-miR-122) and human (hsa-miR-122-5p) sequences (Table 2). Validation of miR-122 expression was conducted by qPCR using the hsa-miR-122-5p TaqMan small RNA assay kit. Expression of miR-122 was up-regulated (*P* < 0.0001) by 217-fold at d 92 and 20.7-fold at d 124 compared to d0 values (Figure 2). These results confirm the miRNA sequencing results in that miR-122 was up-regulated at d 92 by a greater fold change than d 124. The magnitude of the fold change as determined by qPCR is higher than small RNA sequencing which may be related to normalization procedures used for sequencing (transcripts per million) vs. for qPCR (C_T_ values for d0). Predicted targets of miR-122 (glycogen synthase 1 [GYS1], myocyte enhancer factor 2D [MEF2D] and forkhead box O3 [FOXO3]) were down-regulated at d 92 and up-regulated at d 124 (Figure 3).

**Table 4:** Differentially expressed miRNA in skeletal muscle biopsies at d92 on concentrates compared to d0.

**Gene ID**	***P*-Level**	**Regulation**	**Log FC^**1**^**	**miRBase**
bta-miR-122	0.0048	up	6.719	MI0005063
				
bta-miR-323	0.0008	down	−1.018	MI0009797
bta-miR-449a	0.0022	down	−1.406	MI0009834
bta-miR-1197	0.0312	down	−1.451	MI0010470
bta-miR-485	0.0002	down	−1.501	MI0009842
bta-miR-2411	0.0176	down	−1.647	MI0011456

*Log FC = log fold change From d 0*.

**Table 5:** Differentially expressed miRNA in skeletal muscle biopsies at d 124 on concentrates compared to d0.

**Gene ID**	***P*-Level**	**Regulation**	**Log FC^**1**^**	**miRBase**
bta-miR-122	0.0268	up	3.832	MI0005063
bta-miR-383	0.0437	up	1.702	MI0009823
bta-miR-2346	0.0265	up	1.505	MI0011374
bta-miR-144	0.0396	up	1.329	MI0009744
bta-miR-505	0.0126	up	1.258	MI0009856
bta-miR-142	0.0203	up	1.169	MI0005011
bta-miR-1248–1	0.0271	up	1.089	MI0010477
bta-miR-1248–2	0.0290	up	1.043	MI0010483
				
bta-miR-196b	0.0496	down	−1.059	MI0009768
bta-miR-208b	0.0010	down	−1.133	MI0009774
bta-miR-196a-2	0.0243	down	−1.246	MI0009766
bta-miR-362–5p	0.0120	down	−1.292	MI0009811
bta-miR-196a−1	0.0246	down	−1.327	MI0009767
bta-miR-449a	0.0114	down	−1.332	MI0009834
bta-miR-2411	0.0280	down	−1.561	MI0011456

*Log FC = log fold change From d 0*.

**Figure 4:**
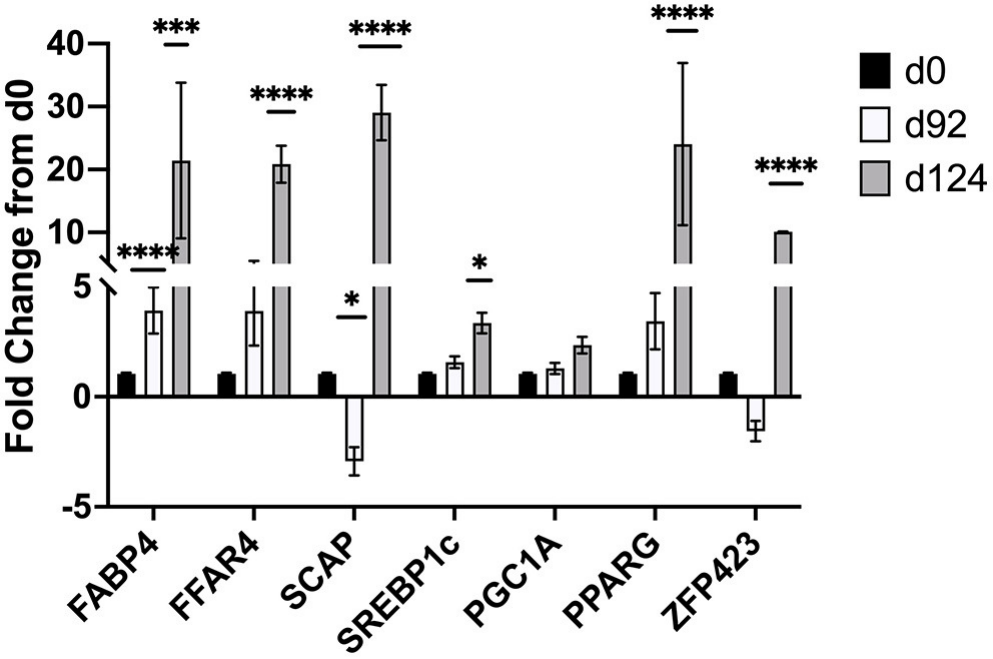
Fold change in mRNA expression of transcription factors (ZFP423, PPARG, SREBP1c), activators (PGC-1A, SCAP), and fatty acid transporters (FABP4, FFAR4) at d 92 and d 124 compared to d 0 (*n* = 4/time). Differences are noted (*P < 0.05; **P < 0.01; ***P < 0.001; ****P < 0.0001) in comparison to d 0.

**Figure 5:**
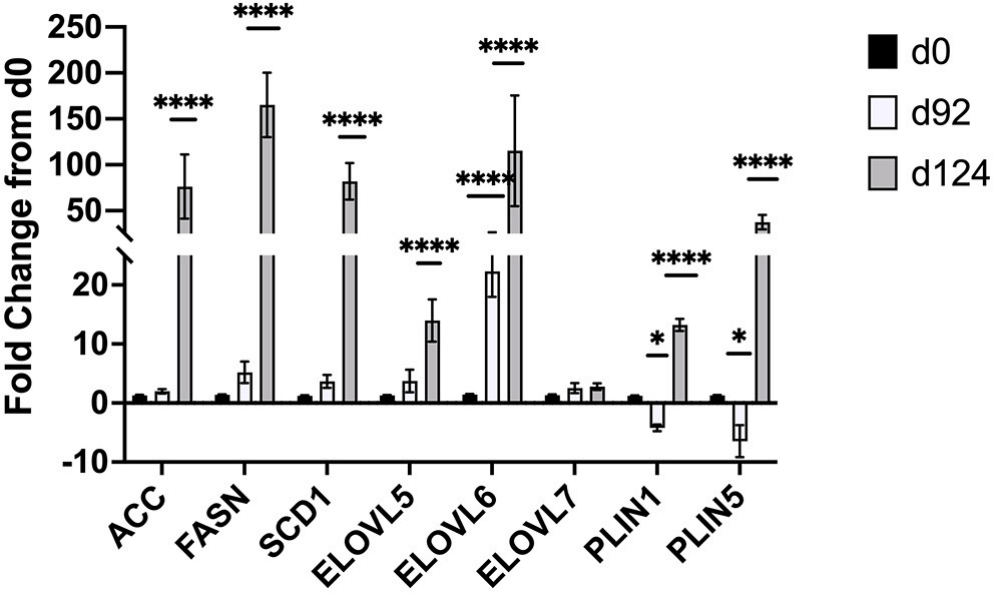
Fold change in mRNA expression of lipogenic (ACC, FASN, SCD1, ELOVL5,6,7) and lipolytic (PLIN1,5) genes at d 92 and d 124 compared to d 0 (*n* = 4/time). Differences are noted (*P < 0.05; **P < 0.01; ***P < 0.001; ****P < 0.0001) in comparison to d 0.

Changes in mRNA expression of adipogenic and lipogenic genes was assessed by qPCR. At d 92 TOC, mRNA expression of fatty acid binding protein 4 (FABP4; Figure 4) and fatty acid elongase 6 (ELOVL6; Figure 5) was up-regulated (*P* < 0.0001) compared to d 0. Expression of other adipogenic genes (peroxisome proliferator-activated receptor γ [PPARG], PPAR γ coactivator-1 α [PGC1A], sterol regulator element binding protein 1c [SREBP1c], and zinc finger protein 423 [ZFP423]), fatty acid transporters (fatty acid binding protein 3 [FABP3], free fatty acid receptor 4 [FFAR4]), and lipogenic genes (acetyl-CoA carboxylase [ACC], fatty acid synthase [FASN], stearoyl-CoA desaturase [SCD1], fatty acid elongase [ELOVL5], fatty acid elongase [ELOVL7]) did not differ between d 92 and d 0 biopsy samples. Perilipin (PLIN1 and PLIN5) and sterol regulatory element-binding protein (SREBP1c) cleavage-activating protein (SCAP) mRNA expression was down-regulated (*P* < 0.05) at d 92 of concentrate feeding compared to d 0. After 124 d on concentrates, lipogenic genes involved in *de novo* fatty acid synthesis (ACC, FASN), fatty acid transport (FABP4, FFAR4), elongation (ELOVL5, ELOVL6) and desaturation (SCD1) were highly up-regulated (*P* < 0.0001) compared to d0 (Figure 5). Zinc finger protein 423 (ZFP423), PPARG, SREBP1c, and SCAP expression were also up-regulated (*P* < 0.05) at d 124 compared to d 0 (Figure 4). Expression of PLIN1 and PLIN5 was up-regulated (*P* < 0.0001) at d 124 compared to d 0.

## Discussion

In this study, we examined changes in growth and feed efficiency of steers fed a high concentrate (90% concentrate) diet over TOC. Steer body weight gains did not change during the initial 28 d on feed when they were training to use Calan gates and adjusting to the high concentrate diet. After 28 d, steer BW increased (*P* < 0.0001) at each period (28 to 33 d) across TOC. Steers finished with an average BW of 659 kg after feeding for 124 d. Average daily gain was the greatest during period 2 (d 29–61) when steers were likely exhibiting compensatory growth phase after lower gains in period 1 (d 0–28). Average daily gains were lower during period 4 (d 93–124) compared to period 2 when steers were increasing in external fat deposition and reaching final BW. The steers in this study were very efficient during periods 1 and 2 with gain:feed at 0.22. Ultrasound fat thickness deposition increased from d 28 to 124 at a rate of 1.36 mm per day. Ribeye area increased across TOF with greater ribeye area at d 61 compared to d 0 and 28 and at d 124 compared to d 61. Intramuscular fat content, as measured by real-time ultrasound, increased on average by 37% between d 61 and 92, and remained constant to d 124. Numerous serial slaughter studies have also observed similar changes in intramuscular fat content after feeding concentrates for 80 to 120 d (3–6).

The average level of intramuscular fat content as measured by ultrasound was 10.03%, which would correspond to the moderately abundant marbling score and Prime quality grade (21). Overall, six of the seven steers evaluated for ultrasound IMF at d 92 and d124 would have been above minimum level of 8.0% IMF for the slightly abundant marbling score and Prime quality grade (21); in contrast, only one steer would have reached this minimum level of IMF (8%) at d 62 on concentrates. External fat thickness increased linearly with time-on-concentrates and the steers in this study had excessive levels at the end of the 124 d feeding period. The use of real-time ultrasound to monitor changes in IMF over time allows us to identify key miRNA and mRNA that are associated with enhanced IMF deposition across TOC. Previous research has shown that the real-time ultrasound IMF estimates are highly correlated to actual longissimus muscle lipid content (9, 10).

Small RNA sequencing identified bta-miR-122 as a potential miRNA of interest that may be associated with intramuscular lipid deposition in the bovine. miR-122 was up-regulated on d 92 (6.72-fold change) and d 124 (3.83-fold change) at the same time when ultrasound IMF levels were also above d 0 values. These results were confirmed by qPCR that miR-122 was up-regulated at d 92 and d 124 compared to d 0, and that up-regulation was greater at d 92. In humans, miR-122 is highly abundant and accounts for 70% of all miRNAs present in the liver. Research shows that miR-122 regulates cholesterol and fatty acid metabolism in humans (22) and tilapia (23–25). In pigs, miR-122 was identified as one of three miRNAs that were regulators of fat deposition (26) and appear to be associated with pyruvate kinase in subcutaneous adipose tissues. Not much is known about miR-122 in the bovine but others (27) have also identified miR-122 as an important miRNA along with two others (miR-381 and miR-499) that are involved with intramuscular fat deposition in the Yak (*Bos grunniens*). However, they observed that miR-122 was down-regulated in LM and adipose tissue from 0.5 yr to 2.5 yr of age. In this study, we only examined changes in miRNA transcriptome during a short interval (124 d; 12.3 to 16.6 mo of age) that coincided with increased intramuscular fat deposition due to feeding high concentrate diets.

TargetScan 8.0 (cow; http://www.targetscan.org/vert_80/) and miRDB (human; http://mirdb.org/) programs were used to identify potential targets for miR-122 due to its potential role in intramuscular fat deposition. TargetScan predicted 194 targets and miRDB predicted 490 targets. From these lists, we selected genes that are known to be involved in muscle or lipid metabolism and examined differences in relative gene expression by TOC using qPCR. Glycogen synthase 1 (GYS1) was identified in both programs as a target with high score for miR-122 in human (target score 95, miRDB) and bovine (-0.96 cumulative weighted context++ score; TargetScan8.0). Additional predicted targets for miR-122 included forkhead box O3 (FOXO3) and myocyte enhancer factor 2D (MEF2D). Our results found that miR-122 and mRNA expression of predicted targets (GYS1, FOXO3, and MEF2D) were inversely related. Expression levels of these targets were down-regulated when miR-122 expression was up-regulated by 217-fold at d 92 and up-regulated when miR-122 expression was up-regulated by only 3.8-fold at d124. Song et al. (28) has shown that miR-122 directly targets FOXO3 in cardiomyocytes undergoing hypertrophy. They found that miR-122 negatively regulated FOXO3, which would agree with our results in that miR-122 was highly expressed at d 92 and FOXO3 was down-regulated. MEF2D was confirmed as a miR-122 target in cardiac myxoma cells (29). They reported that PPARG and MEF2D are inversely related and that up-regulation of miR-122 activates PPARG to inhibit MEF2D, which reduces proliferation of cardiac myxoma cells. In order to examine the role of miR-122 in lipid metabolism, Esau and co-workers used an antisense oligonucleotide (ASO) treatment in mice to inhibit miR-122 (30). They found increased expression of GYS1 in the liver tissue of the miR-122 ASO treated mice in a dose-response manner. They also examined changes in lipogenic genes in the liver by microarray and found that miR-122 inhibition down regulated FASN, ACC, and SCD1 expression by about 1.5 to 3-fold change. These authors postulated that miR-122 may alter expression of a transcriptional inhibitor because the seed sequence for miR-122 is not predicted to bind to these lipogenic genes according to the available algorithms (TargetScan, miRDB or others). Miravirsen (SPC3649) can inhibit miR-122 biogenesis and is the first anti-miRNA ASO to enter clinical trials for hepatitis C virus treatment in humans (31). Additional research is needed to determine how miR-122 may be involved in lipogenesis/lipolysis and to determine if miR-122 mimics or enhancers could be used to stimulate intramuscular fat deposition.

There were a few miRNAs (miR-323, −449a,−1197, −485) that were down-regulated at d 92 but they had a low fold change (< −1.7) and no established role in adipose or muscle metabolism. On d 124 TOC, there were other miRNAs that were up or down regulated compared to d 0. Most of these miRNAs (miR-383, 2,346, 505, 1,248, 196, 208, 362, or 449) have no known role in adipose or muscle metabolism. miR-2411 was down-regulated at both d 92 and 124 compared to d 0. Little is known about miR-2411 but it was identified as a novel miRNA in subcutaneous fat of pigs and Meishan had higher expression than Large White pigs but expression levels were low in abundance for both breeds (32). miR-142, −144 and −196 appear to be involved with lipid metabolism and insulin resistance. miR-144 binds to insulin receptor substrate (IRS1) to control its expression and appears to be a potential therapeutic target for type 2 diabetes treatment in humans (33, 34). Muroya and coworkers (35) examined plasma exosomal miRNAs and found that miR-142–5p was down-regulated in cattle after grazing for 3 mo. In subcutaneous fat, miR-142–5p expression was greater in grazing cattle and may be related to fatty acid metabolism (14, 35). miR-196a was identified in swine back fat samples as having a tissue specific expression pattern with mature animals having highest levels of expression in subcutaneous fat and liver (36). *In vitro* experiments that overexpressed miR-196a showed that it stimulated preadipocyte differentiation but did not alter proliferation (36). In our study, miR-142 and miR-144 were up regulated (*P* < 0.05) and miR-196a-1 and miR-196a-2 were down regulated at 124 d on concentrates.

At d 92 of TOC, there was up-regulation of fatty acid binding protein 4 (FABP4, 4-fold change) and fatty acid elongase 6 (ELOVL6; 8-fold change) mRNA expression, and down-regulation of mRNA involved in lipolysis (PLIN1 and PLIN5) and adipogenesis (SCAP) compared to d 0. FABP4 transports intracellular fatty acids to the nucleus where it alters transcription of certain genes (37). FABP4 expression is up-regulated during adipocyte differentiation (38) and serves as a marker of differentiation in bovine adipocytes (37, 39). Michal et al. (40) reported that FABP4 was associated with subcutaneous fat thickness and marbling in Wagyu x Limousin F_2_ cattle. Guo and co-workers (41) related gene expression to IMF percentage in cattle and sheep and identified FABP4 as having a significant association with IMF in both species. The up-regulation of FABP4 with greater TOC indicates that concentrate finishing may enhance uptake of fatty acids into the nucleus and promote differentiation of preadipocytes.

Fatty acid elongase 6 (ELOVL6) is involved in the elongation of palmitic (C16:0) acid to stearic (C18:0) acid (42, 43). Knockdown of ELOVL6 in rat insulinoma cell lines (42) and mice (43) demonstrated that ELOVL6 is required for monounsaturated fatty acid synthesis. These results show that ELOVL6 plays a pivotal role in monounsaturated fatty acid synthesis, which is the one of the main fat types in beef muscle and increased concentrations are observed with high concentrate feeding (3, 11). In this study, the up-regulation of ELOVL6 at d 92 precedes the up-regulation of lipogenic genes involved in *de novo* fatty acid synthesis (ACC, FASN) and desaturation (SCD1) observed at d124. Perilipins are associated with intracellular lipid droplets where they stabilize the droplet and limit access by cytosolic lipases thereby regulating triacylglyceride storage under basal conditions (44). Perilipin 1 (PLIN1) is most abundant in white adipose tissues where it is involved with hormone-stimulated lipolysis; whereas perilipin 5 (PLIN5) is expressed in cardiac and skeletal muscle, and brown adipose tissues where it regulates fatty acid supply to the mitochondria (45). In pigs, immunohistochemistry was used to determine where PLIN proteins were located within the longissimus muscle of low or normal birth weight piglets at specific days (5, 12, and 26 d) postnatal (46). PLIN3 and PLIN4 were found at the periphery of muscle fibers and intramuscular adipocytes; in contrast, PLIN5 was localized within an undefined cell type located between the muscle fibers (46). Sterol regulatory element-binding protein (SREBP1c) cleavage-activating protein (SCAP) is required to activate all isoforms of SREBP. In mice, knockdown of SCAP reduces expression of genes involved in cholesterol and fatty acid synthesis by 70-80% in the liver (47). Others have also identified ELOVL6 and PLIN5 as being of high importance in bovine adipose tissues in the Yak (48). Nakajima et al. (49) identified PLIN5 in a genome-wide association study in Japanese Black cattle with high intramuscular fat deposition.

At 124 d of TOC, lipogenic genes involved in *de novo* fatty acid synthesis (ACC, FASN), fatty acid transport (FFAR4, FABP4), elongation (ELOVL5, ELOVL6) and desaturation (SCD1) were highly up-regulated compared to d 0. Other genes involved in lipolysis (PLIN1 and PLIN5) and transcription (ZFP423, PPARG, SREBP1c, SCAP) were up-regulated at d 124 compared to d0. Previous research has also shown up-regulation of FASN and SCD1 in subcutaneous fat from steers fed high concentrate diets compared for forage-finished (11). Others have shown that feeding high concentrates to steers upregulates key lipogenic genes and marbling deposition in early weaned calves (7, 8), normal weaned calves (9, 10) and yearling calves (11). Graugnard and co-workers (8) reported that feeding high starch diets to early-weaned steers up-regulated lipogenic genes (FASN, FABP4, SCD1, PPARG and PGC1A) at d 56 to stimulate differentiation of preadipocytes; whereas feeding low starch diets delayed the up-regulation of lipogenic genes (FABP4, FASN, SCD, DGAT2) until 112 d. Up-regulation of transcription factors (ZFP423, PPARG, SREBP1c, SCAP) at d 124 on concentrates suggests that another wave of proliferation may be occurring. Robelin (50, 51) examined changes in fat deposition from 15 to 65% of mature weight in cattle and reported that fat deposition began with an increase in cell number (hyperplasia) followed by the filling of these cells (hypertrophy), which then stimulated another increase in hyperplasia that occurred at 45 to 55% of mature weight.

## Conclusions

The results of this study identified bta-miR-122 as a potential miRNA of interest that may be involved in intramuscular fat deposition with increasing time-on-concentrates. More research is needed to further define the role of miR-122 but potential target genes were identified and differentially expressed. Changes in mRNA expression show that FABP4, ELOVL6, PLIN1 and PLIN5 are differentially expressed at d 92 prior to the up-regulation of lipogenic genes involved in *de novo* fatty acid synthesis at d 124. The greater intramuscular fat content as measured by real-time ultrasound at d 92 combined with differential gene expression suggest that preadipocyte differentiation may be promoted at this stage of TOC, which is followed by a global up-regulation of key lipogenic genes, fatty acid transporters, and desaturases that provide fatty acids for adipocyte hypertrophy.

## Data Availability Statement

The datasets presented in this study can be found in online repositories. The names of the repository/repositories and accession number(s) can be found below at https://www.ncbi.nlm.nih.gov/geo/query/acc.cgi?acc=GSE197315.

## Ethics Statement

The animal study was reviewed and approved by Experimental procedures were reviewed and approved by Clemson University Animal Care and Use Committee, AUP2020-001.

## Author Contributions

SD designed the research project, conducted skeletal muscle biopsies and molecular analyses. MG assisted with miRNA sequencing design and data analyses. SD drafted the manuscript and MG assisted with editing. Both authors contributed to the article and approved the submitted version.

## Funding

Technical contribution No. 7036 of Clemson University Experiment Station. This material is based upon work supported by NIFA/USDA, under project number SC-1700580.

## Conflict of Interest

The authors declare that the research was conducted in the absence of any commercial or financial relationships that could be construed as a potential conflict of interest.

## Publisher's Note

All claims expressed in this article are solely those of the authors and do not necessarily represent those of their affiliated organizations, or those of the publisher, the editors and the reviewers. Any product that may be evaluated in this article, or claim that may be made by its manufacturer, is not guaranteed or endorsed by the publisher.
