# Supplementary material for: Identification of microRNA Transcriptome Involved in Bovine Intramuscular Fat Deposition
Source: Front Vet Sci. 2022 Apr 15;9:883295. doi: 10.3389/fvets.2022.883295 (PMC9051433; doi:10.3389/fvets.2022.883295)
Supplement: Supplementary file 1 [file Table_1.DOCX]

**Supplemental Table S1.** Primer sequences (5’ to 3’) for quantitative real-time PCR of key genes in adipogenesis and muscle development.

| Gene^1^ | Forward | Reverse | Efficiency |
| --- | --- | --- | --- |
| ACC | AGCTGAATTTTCGCAGCAAT | GGTTTTCTCCCCAGGAAAAG | 1.07 |
| ACTB | CTCTTTCAGCCTTCCTTCCT | GGGCAGTGATCTCTTTCTGC | 1.01 |
| EIF3K | CTGACAGACAGCCAGCTAAA | CACGATGTTCTTGGGCTTTATG | 1.02 |
| ELOVL5 | GTCATCTGGCCGTGTACCTT | GGGAAGAAAAGCTGCTGATG | 1.02 |
| ELOVL6 | GGAAAGCAACGAAAGCTGAC | TGGGTTGTGTGTTTGCTCAT | 1.05 |
| ELOVL7 | CGACTTTCACTCACTTGGTATCT | CTCTGCCAGCTGTGATTTCT | 1.01 |
| FABP3 | TATGACCAAGCCTACCACAATC | TCACGATGGACTTGACTTTCC | 1.02 |
| FABP4 | CATCTTGCTGAAAGCTGCAC | AGCCACTTTCCTGGTAGCAA | 1.00 |
| FASN | GCATCGCTGGCTACTCCTAC | GTGTAGGCCATCACGAAGGT | 0.95 |
| FFAR3 | ACTCCTTCTTCCTCGGCAAT | AGATCCGAGAGGGTGAGGTT | 1.05 |
| FFAR4 | AGGAACGAATGGAGGAGGTT | GTGCTGAGGGTCATGGAAAT | 1.04 |
| FOXO3 | CCAGTCTATGCAAACCCTCTC | CCATGAGTTCGCTACGGATAAT | 1.00 |
| GAPDH | GGGTCATCATCTCTGCACCT | GGTCATAAGTCCCCTCCACGA | 0.97 |
| GYS1 | TCCAGAGAATGGATGTGGAATG | GGAACTCCAGGGCACAATAA | 1.01 |
| MEF2D | TGTGTCTCTCAGCAACCTAATC | ACACTGGTTCCGACTTGATG | 1.00 |
| PLIN1 | CCCAGTCCAGGAGACAATAGA | ACCAAGGACCAAACCTGTATC | 1.00 |
| PLIN5 | AAGTCAGAGGAGCTGGTGGA | CCCAGAGAGTGCTCATAGGC | 1.00 |
| PPARG | AGGATGGGGTCCTCATATCC | GCGTTGAACTTCACAGCAAA | 1.00 |
| PGC-1A | CTTCCTCCTGACACCCATGT | CGCTCCTCAGAAAGAACCAC | 0.98 |
| SCAP | GGCTGATCCATGGTCACTTT | AGTGGGTAGCAGCAGGCTAA | 1.05 |
| SCD1 | TTATTCCGTTATGCCCTTGG | GGTAGTTGTGGAAGCCCTCA | 0.95 |
| SREBP1c | CTGGAGAAGCTGGACTGAGG | GCTTTCCCAAGACTCAGCAC | 0.95 |
| UXT | GGTGGATTTGGGCTGTAACT | TGTGGATATGGGCCTTGATATTC | 1.00 |
| ZFP423 | GAAGACCATCCACGCAGATAAG | GATGCTGCCAAACTGCATTAC | 1.00 |

^1^Acetyl CoA carboxylase (ACC); beta-actin (ACTB); eukaryotic translation initiation factor 3 (EIF3K); fatty acid elongase 5 (ELOVL5); fatty acid elongase 6 (ELOVL6); fatty acid elongase 7 (ELOVL7); fatty acid synthase (FASN); fatty acid binding protein 3 (FABP3); fatty acid binding protein 4 (FABP4); free fatty acid receptor 3 (FFAR3); free fatty acid receptor 4 (FFAR4); forkhead box O3 (FOXO3); glyceraldehyde 3-phosphate dehydrogenase (GAPDH); glycogen synthase 1 (GYS1); myocyte enhancer factor 2D (MEF2D); perilipin 1 (PLIN1); perilipin 5 (PLIN5); peroxisome proliferator-activated receptor γ (PPARG); PPARγ coactivator-1 α (PGC-1A); SREBP cleavage activating protein (SCAP); stearoyl CoA desaturase-1 (SCD1); sterol regulatory element-binding protein 1c (SREBP1c); Ubiquitously expressed prefoldin like chaperone (UXT); zinc finger protein 423 (ZFP423).
